# *Plasmodium vivax* populations in the western Greater Mekong Subregion evaluated using a genetic barcode

**DOI:** 10.1371/journal.pntd.0012299

**Published:** 2024-07-03

**Authors:** Yubing Hu, Yuling Li, Awtum M. Brashear, Weilin Zeng, Zifang Wu, Lin Wang, Haichao Wei, Myat Thu Soe, Pyae Linn Aung, Jetsumon Sattabongkot, Myat Phone Kyaw, Zhaoqing Yang, Yan Zhao, Liwang Cui, Yaming Cao

**Affiliations:** 1 Department of Immunology, College of Basic Medical Sciences, China Medical University, Shenyang, Liaoning, China; 2 Emergency Department, The First Affiliated Hospital of Dalian Medical University, Dalian, Liaoning, China; 3 Division of Infectious Disease and International Medicine, Department of Internal Medicine, Morsani College of Medicine, University of South Florida, Tampa, Florida, United States of America; 4 Department of Pathogen Biology and Immunology, Kunming Medical University, Kunming, China; 5 Myanmar Health Network Organization, Yangon, Myanmar; 6 Mahidol Vivax Research Unit, Faculty of Tropical Medicine, Mahidol University, Bangkok, Thailand; SUCEN/IMT/USP, BRAZIL

## Abstract

An improved understanding of the *Plasmodium vivax* populations in the Great Mekong Subregion (GMS) is needed to monitor the progress of malaria elimination. This study aimed to use a *P*. *vivax* single nucleotide polymorphism (SNP) barcode to evaluate the population dynamics and explore the gene flow among *P*. *vivax* parasite populations in the western GMS (China, Myanmar and Thailand). A total of 315 *P*. *vivax* patient samples collected in 2011 and 2018 from four regions of the western GMS were genotyped for 42 SNPs using the high-throughput MassARRAY SNP genotyping technology. Population genetic analysis was conducted to estimate the genetic diversity, effective population size, and population structure among the *P*. *vivax* populations. Overall, 291 samples were successfully genotyped at 39 SNPs. A significant difference was observed in the proportion of polyclonal infections among the five *P*. *vivax* populations (*P* = 0.0012, Pearson Chi-square test, *χ*^*2*^ = 18.1), with western Myanmar having the highest proportion (96.2%, 50/52) in 2018. Likewise, the average complexity of infection was also highest in western Myanmar (1.31) and lowest in northeast Myanmar (1.01) in 2018. The older samples from western China in 2011 had the highest pairwise nucleotide diversity (*π*, 0.388 ± 0.046), expected heterozygosity (*He*, 0.363 ± 0.02), and the largest effective population size. In comparison, in the neighboring northeast Myanmar, the more recent samples in 2018 showed the lowest values (*π*, 0.224 ± 0.036; *He*, 0.220 ± 0.026). Furthermore, the 2018 northeast Myanmar parasites showed high and moderate genetic differentiation from other populations with *F*_ST_ values of 0.162–0.252, whereas genetic differentiation among other populations was relatively low (*F*_ST_ ≤ 0.059). Principal component analysis, phylogeny, and STRUCTURE analysis showed that the *P*. *vivax* population in northeast Myanmar in 2018 substantially diverged from other populations. Although the 42 SNP barcode is a valuable tool for tracking parasite origins of worldwide parasite populations, a more extended barcode with additional SNPs is needed to distinguish the more related parasite populations in the western GMS.

## Author summary

In the Great Mekong Subregion (GMS), particularly in Myanmar, vivax malaria remains a significant challenge to malaria elimination. To effectively evaluate the impact of ongoing malaria control measures, it is essential to understand the genetic diversity, relatedness, and population dynamics of the malaria parasite. A comprehensive analysis of *P*. *vivax* populations in the western GMS using a global 42-SNP barcode revealed notable changes over time. Compared to the more homogeneous parasite populations a decade ago, there has been a decrease in genetic diversity and an increase in differentiation among parasite populations in recent years, particularly along the China-Myanmar border. In comparison, the 2018 parasites from western Myanmar showed a relatively stable genetic structure, underscoring the persistent challenge of vivax malaria in this region. While the 42-SNP barcode has been valuable in understanding the genetic landscape of global *P*. *vivax* populations, it has limitations in accurately differentiating parasite populations across the GMS, necessitating a barcode tailored to the local parasite populations.

## Introduction

*Plasmodium vivax* is the most widely distributed species of human malaria and is endemic in Asia, Central and South America, the Middle East, Oceania, and East Africa. In 2021, about 4.94 million malaria cases were caused by *P*. *vivax* worldwide, with 42% of the burden in the Southeast Asia Region [1]. Compared with *P*. *falciparum*, *P*. *vivax* is more resilient to conventional control measures due to the production of dormant hypnozoites responsible for relapses, low-density infections evading detection, and the early emergence of gametocytes favoring transmission [2,3]. Evidence that *P*. *vivax* can cause severe malaria and deaths continues to mount [4]. Further, *P*. *vivax* has become increasingly resistant to chloroquine, the first-line treatment in most vivax-endemic countries [5]. Accordingly, *P*. *vivax* has become the predominant malaria parasite in many endemic regions, demanding more effective strategies for its control and elimination.

Various genetic markers have been used to differentiate parasite populations, study transmission dynamics, examine epidemic events such as population decline and expansion, monitor the spread of drug-resistant parasites, and track possible infection sources and routes of introduction [6–9]. Microsatellite markers have been widely used for such analyses [10–14], and an open-access platform, VivaxGEN, has been established to standardize microsatellite-based population genetic analysis in *P*. *vivax* [15]. Advances in the next-generation sequencing technologies allowed whole-genome sequencing (WGS) of parasites from world populations of *P*. *falciparum* [16,17] and *P*. *vivax* [18,19], starting the new era of malaria population genomics, when parasite population structure, transmission dynamics and complexity of infection can be accurately elucidated at the whole-genome level [20, 21]. The large number of single nucleotide polymorphisms (SNPs) uncovered from the WGS of the global malaria parasite populations has enabled the selection of sets of SNP markers as “genetic barcodes” to study the parasite transmission networks and population dynamics. Compared to microsatellite panels, SNP barcodes are easier to standardize and can more accurately detect genetic diversity between populations and geographic population structures [22]. SNP barcodes have been successfully applied to determine population transmission patterns of *P*. *falciparum* in different regions of the African continents [6, 23–26]. Similarly, a 42-SNP barcode was proposed for the *P*. *vivax* parasites based on relatively limited genome datasets [27]. A more comprehensive barcode of 71 SNPs was recently designed based on a significantly expanded *P*. *vivax* WGS dataset, which was shown to correctly predict the geographic origins of the parasites [28]. In addition, region-specific barcodes aiming to resolve parasite populations within a region have been designed [29,30]. More recently, a panel of 100 microhaplotypes, each containing multiple high-frequency SNPs, was designed to better resolve the global *P*. *vivax* populations using an amplicon-sequencing approach [31]. To date, the *P*. *vivax* SNP barcodes have only been used in a limited number of studies [22,27,32], while their utility in evaluating parasite population dynamics remains to be vigorously tested.

Countries of the Greater Mekong Subregion (GMS) in Southeast Asia are pursuing regional malaria elimination by 2030 [33]. Within this region, Myanmar has the highest malaria burden [34]. Like in other countries of the GMS, the proportion of malaria caused by *P*. *vivax* in Myanmar has increased steadily since 2012 [35,36]. As the malaria elimination course progressed, malaria transmission became concentrated along international borders and in disconnected regions. Because of the disparity of malaria incidence among regions and between the two sides of the border and extensive cross-border migration [37], it is critical to understand the genetic differences of parasite populations so that sources of imported and migration-related malaria can be traced. Studies using microsatellite markers have detected substantial geographical differentiation and temporal changes in parasite populations in the western GMS [38–40], whereas population genomics revealed a more homogeneous parasite population in the eastern GMS, Cambodia and Vietnam [30,41]. Herein, we evaluated the 42-SNP barcode to differentiate *P*. *vivax* populations in the western GMS, focusing on Myanmar and its adjacent areas in the Tak Province of Thailand and Yunnan Province of China.

## Methods

### Ethics approval

The study protocols were approved by institutional ethical committees of the collaborating institutions. Ethical approval was obtained from the institutional review boards of China Medical University, China (2019086), University of South Florida, USA (Pro00036813), Mahidol University, Thailand (TMEC11-033), and the Ministry of Health and Sports, Myanmar (Ethics/DMR/2017/077AE/2018). Written informed consent was obtained from all adult participants or the legal guardians of children before conducting the study.

### Study sites and sample collection

**Fig 1:**
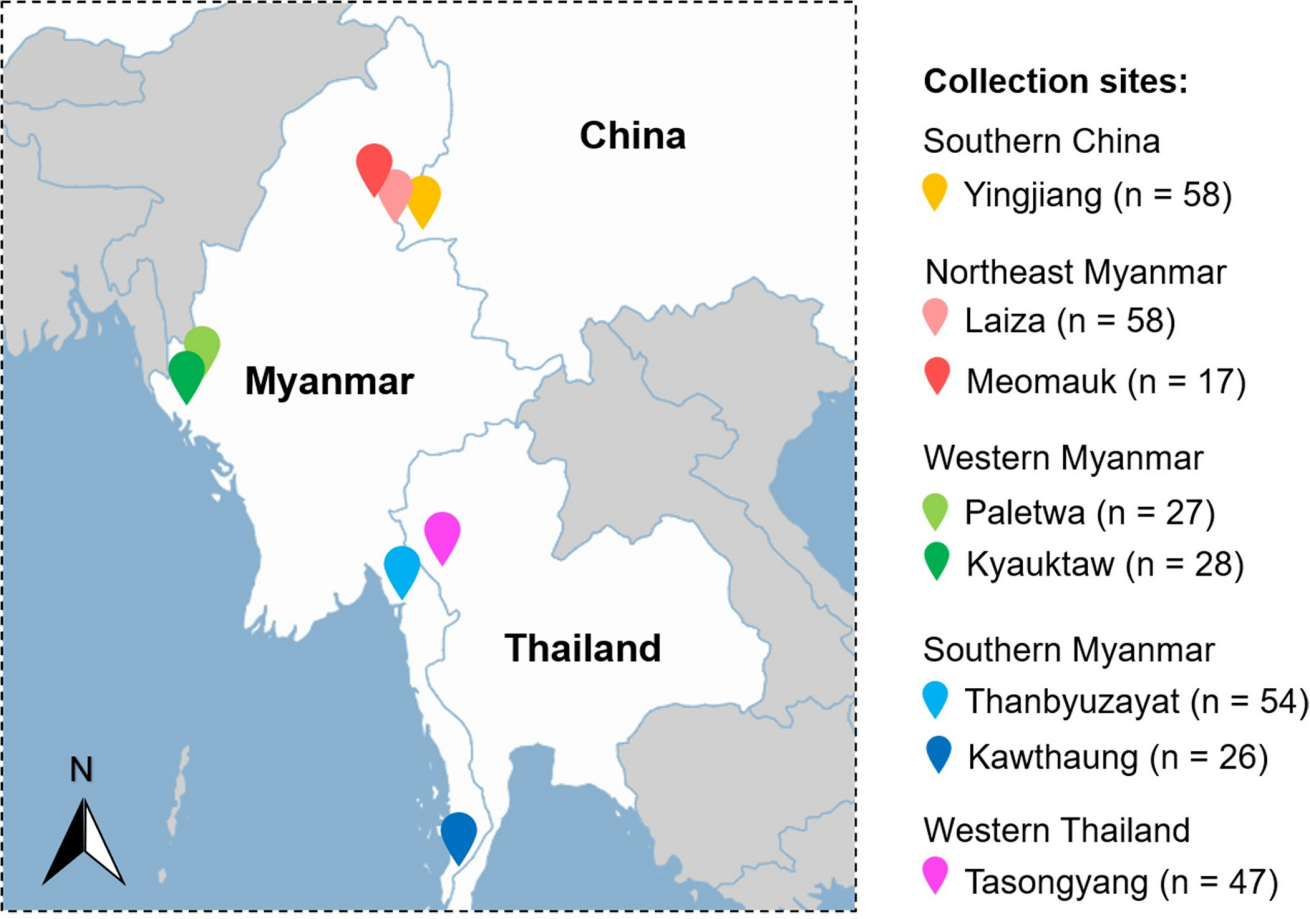
Map of study sample collection sites in the Great Mekong Subregion. Specific collection sites and sample sizes for SNP barcode analysis are represented by landmarks with different colors. The map was generated using the online tool available at https://pixelmap.amcharts.com/. The license information can be accessed at https://www.amcharts.com/licenses/amcharts-20240401/.

*P*. *vivax* clinical samples were collected from patients with uncomplicated vivax malaria diagnosed by microscopy at local malaria clinics or hospitals in western China (WC2011, Yingjiang, Yunnan province, 2011, n = 58), northeast Myanmar (NEM2018, Laiza and Meomauk, 2018, n = 75), western Myanmar (WM2018, Kyauktaw and Paletwa, 2018, n = 55), and southern Myanmar (SM2011, Thanbyuzayat and Kawthaung, 2011, n = 80). Samples were also collected from bordering areas of western Thailand (WT2011, Mae Sod, Tak province, 2011, n = 47) (Fig 1). After obtaining informed consent (assent from minors), finger-prick blood was spotted on filter papers, which were dried and stored individually in plastic bags with desiccants.

### Genotyping of the 42-SNP barcode

*P*. *vivax* genomic DNA was extracted from dried blood spots on filter papers using the QIAamp DNA minikit (Qiagen). The DNA was eluted into 35–45 μL of elution buffer and typically had an OD 260/280 of 1.6–1.8 and a concentration of > 5 ng/μL as measured using the NanoDrop 2000C spectrophotometer. *P*. *vivax* was confirmed by nested PCR targeting the *P*. *vivax* 18S rRNA using 2 μL of the purified DNA [42].

The 42-SNP barcode was designed to distinguish several global *P*. *vivax* populations. All SNPs are bi-allelic and not located in repetitive sequences, and 18 are in protein-encoding genes [27]. High-throughput genotyping of the 42-SNP barcode was performed on the iPLEX SEQUENOM MassARRAY platform (Sequenom, CA, USA), which uses single-base extension reactions to create allele-specific products that are separated automatically and scored in a matrix-assisted laser desorption ionization/time of flight mass spectrometer. Primer design was performed using MassARRAY Assay Design software (v3.1) according to Sequenom’s instructions (S1 Table). Multiplex PCR amplification of amplicons containing SNPs of interest was performed using the HotStart Taq Polymerase (Qiagen, CA, USA) with 12 ng of genomic DNA. Assay data were analyzed using Sequenom TYPER software (Typer 4.0). Genotypes of the target sites of each sample were interpreted according to the mass spectrograph. Positive and negative controls were included in every panel to ensure genotyping accuracy. Two randomly selected *P*. *vivax* samples from our collection were included as positive quality control, while double-distilled water was used as negative control. Additionally, we assessed the reproducibility of genotyping by (1) subjecting the positive control samples to repeated testing in multiple panels and (2) replicating the results using the MassARRAY and Taqman platforms for samples with multiple biallelic loci. In addition, the previously published SNP barcode data for *P*. *vivax* samples from Africa (n = 15), South America (n = 53), and South Asia (n = 19) were downloaded for comparison [27].

### Data screening and quality filtration

Only ATCG, N (mixed infection), and X (negative or missing SNP) were present in the barcode data. A sample was determined to have mixed infections if peaks matching two control genotypes were observed in at least one locus. A sample was considered negative if it showed no discernable peaks or if its peaks did not match those of any of the controls [25,43].

Minor allele frequency (MAF) was calculated from allele counts for each SNP in each population using PLINK v1.07 [44]. Because sampling was not even across all sites, we also characterized the total MAF across all populations to view regional diversity. For quality filtration, SNPs with a total MAF of less than 5% (—maf 0.05) and individuals with more than 10% missing SNP calls (—mind 0.1) were filtered out [25,43]. In addition, multiallelic SNPs with higher than 5% MAF were also considered polymorphic SNPs. For each polymorphic genotype, we counted calls for both the reference and alternate alleles by designating each with a half contribution compared to monomorphic genotypes.

### Complexity of infection (COI)

Polygenomic (i.e., polyclonal) infections were established in the parasite population by examining the number of heterozygous SNPs in each sample. Since malaria parasites in the human blood are haploid, samples that carried a single allele at all positions were classified as monoclonal infections, while all other samples were considered polyclonal. Regarding samples with only one SNP site with two alleles, we followed the definition proposed by previous study and referred to them as biclonal infections [45].

The most likely number of clones in each infection was estimated with genotypes at the initially targeted SNPs using a maximum likelihood approach implemented in COIL [45,46]. The likelihood method estimated COI from the SNP genotyping data using binomial probability calculations of monomorphic *vs* polymorphic genotype likelihoods produced using the population MAF of the SNPs comprising the barcode panel [46]. The REAL MCCOIL method applied 10,000 iterations of Markov chain Monte Carlo (MCMC) during MAF and COI distribution estimation. It is worth mentioning that only samples with monoclonal and biclonal infection were included in the following analysis of genetic diversity and population differentiation.

### Genetic diversity

To estimate within-population genetic diversity, we calculated the number of haplotypes (*Nh*), the number of alleles (*Na*), the number of effective alleles (*Ae*), and expected heterozygosity (*He*) using GenAIEx version 6.5 [47]. In addition, nucleotide diversity (*π*) was estimated as described [27]. To determine the distribution of genetic variance among the populations, analysis of molecular variance (AMOVA) was performed using GenAlEx version 6.5. The correlation between geographic and genetic distances was calculated using the Mantel rank test in GenAlEx version 6.5.

### Effective population size (*Ne*)

The effective population size was calculated using the stepwise mutation model (SMM) and infinite alleles model (IAM) as previously described [48]. Mutation rates for *P*. *vivax* are lacking, and thus the mutation rate of *P*. *vivax* calibrated from an extinct European strain (5.57 × 10^−7^, 95% CI: 2.75 × 10−8–1.06 × 10^−6^) was used [49].

### Genetic differentiation and population structure analysis

Based on quality-controlled data, we calculated the pairwise *F*_ST_ [50] in five populations using VCFtools [51]. Genetic differentiation was divided into three levels according to the *F*_ST_ value: low (≤ 0.15), moderate (0.15–0.25), and high (≥ 0.25) [52].

Principal component analysis (PCA) was performed by constructing a genetic relationship matrix and estimating eigenvalues and eigenvectors for the principal components using PLINK 1.07 [53]. A two-dimensional scatter plot of the first two principal components was drawn using R (www.rproject.org/). In addition, phylogenetic relationships amongst *P*. *vivax* isolates were analyzed using the Neighbour-Joining method [54] implemented in MEGA7 [55]. The genetic structure of the five populations was analyzed with ADMIXTURE [56]. Ten runs were performed, with K values ranging from 1 to 10, and the one with the lowest cross-validation error was selected.

## Results

### Genotyping the 42-SNP barcode

**Fig 2:**
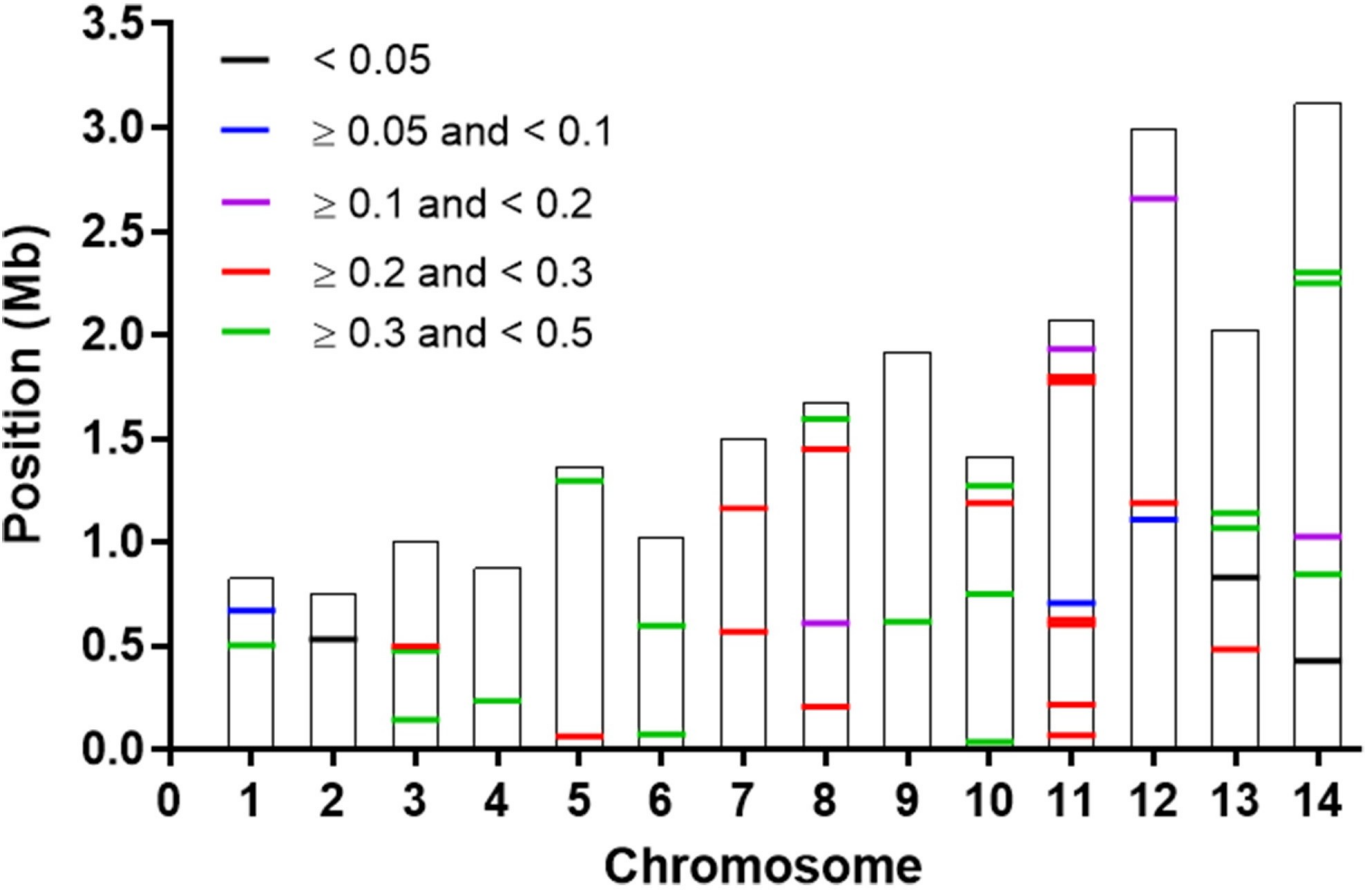
The total minor allele frequency (MAF) of 42 SNPs. The location of the 42 SNPs is illustrated on the 14 chromosomes of the *P*. *vivax* genome. SNPs were colored by total MAF. 17 SNPs had 0.3 ≤ MAF < 0.5, 15 SNPs had 0.2 ≤ MAF < 0.3, 4 SNPs had 0.1 ≤ MAF < 0.2, 3 SNP had 0.05 ≤ MAF < 0.1, 3 SNPs had MAF < 0.05.

We genotyped 315 samples for the 42-SNP barcode in two panels on the MassARRAY platform. To assist SNP calling and ensure accuracy, we included two positive and two negative controls. The peaks corresponding to the reference or alternative bases were consistent in the positive control samples but were either undetected or appeared as minor peaks in negative controls (S1 Fig). The overall genotyping success rate was 97.4% (≥ 94.0% at individual loci) (S1 Data). Based on the filtering criteria, SNPs at positions 530130 on chromosome (chr) 2, 828771 on chr 13, and 427515 on chr 14 with total MAF value below 0.05 were filtered out, leaving 39 SNPs for analysis (S2 Table). Thirty-six SNPs had total MAF above 0.1 (Fig 2 and S2 Table), while SNPs at positions 668364 on chr 1, 705724 on chr 11, and 1108185 on chr 12 had relatively low diversity with total MAF of 0.056, 0.093, and 0.097, respectively (S2 Table). Each sampling site had 35–37 SNPs with MAFs greater than 0.1 (S2 Fig). For SNPs with an MAF greater than 0.3, the WM2018 population had 23 SNPs, and the NEM2018 population had only 15 SNPs (S2 Fig). Meanwhile, 24 samples with more than 10% missing SNP calls (—mind 0.1) were excluded from the analysis. A total of 291 samples were successfully genotyped at 39 SNPs, with only 34 infections (11.7%) exhibiting monoallelicity at each nucleotide position (i.e., monoclonal), while the remaining 257 (88.3%) samples displayed two alleles with ≥ 1 SNP (i.e., polyclonal). Among the latter, 100 infections (38.9%) showed two alleles at a single locus (i.e., biclonal). Ultimately, 134 samples (23 from WC2011, 49 from NEM2018, 14 from WM2018, 25 from SM2011, and 23 from WT2011) with monoclonal and biclonal infections were used for population genetic analyses (S3 Table). The analysis utilized a total of 234 SNP-sequences, with the inclusion of 200 SNP-sequences derived from the generation of 100 biclonal samples.

### Complexity of infection (COI)

**Fig 3:**
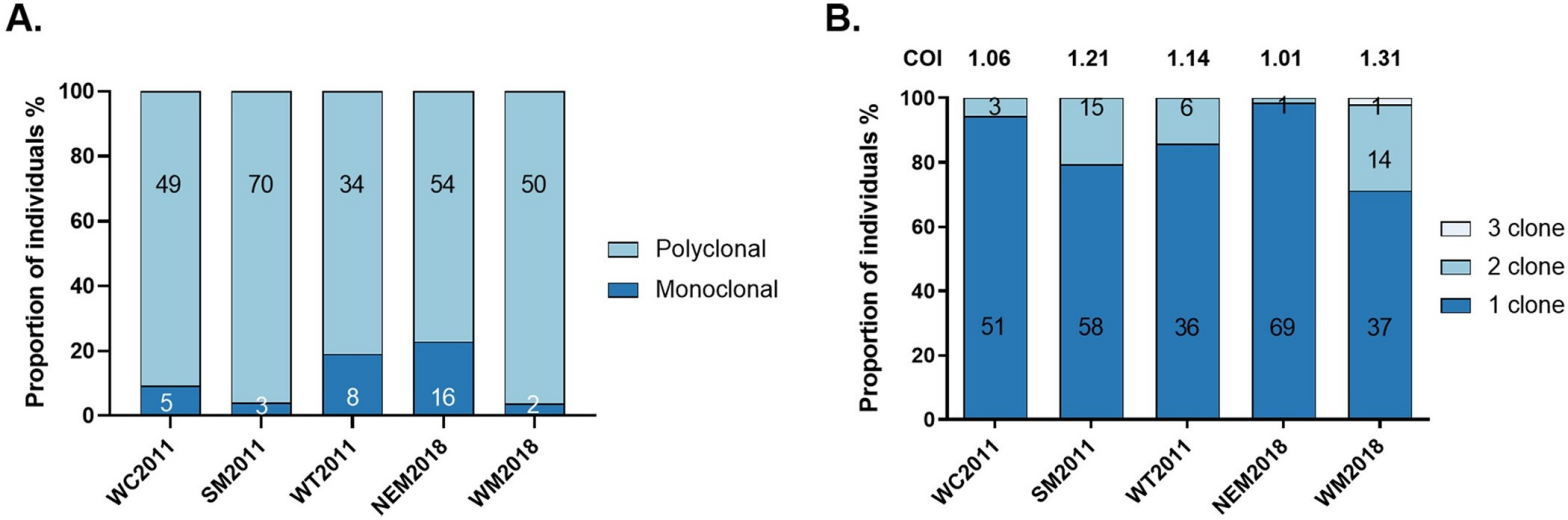
Complexity of infection in different *P*. *vivax* populations in the GMS. (A) Monoclonal and polyclonal infections were calculated using genotypes at the initially targeted SNPs, with samples carring a single allele at all positions being classified as monoclonal infections. (B) The most likely number of clones was estimated using the COIL web-based tool. Numbers within the bars indicate the sampling size for each category. The estimated numbers of polyclonal/monoclonal samples differ between the two methods.

Genotyping results at the 39 SNPs showed that 88.3% of the *P*. *vivax* samples were polyclonal infections. The proportions of polyclonal infections differed significantly among the five *P*. *vivax* populations (*P* = 0.0012, Pearson Chi-square test, *χ*^*2*^ = 18.1), highest (96.2.1%, 50/52) in the 2018 western Myanmar (WM) population and lowest (77.1%, 54/70) in the 2018 northeast Myanmar (NEM) population (WM vs NEM in 2018: *P* = 0.004, Fisher’s exact test) (Fig 3A). Samples collected in 2011 in southern Myanmar had a significantly higher proportion of polyclonal infections than in other two regions (*P* = 0.031, Pearson Chi-square test, *χ*^*2*^ = 6.9). Likewise, the average COI was also highest in WM (1.31) and lowest in NEM (1.01) in 2018 (Fig 3B). In the GMS, we did not observe a significant association between the proportion of polyclonal infections or the most likely number of clones and the year of collection (*P* = 0.196, Fisher’s exact test; *P* = 0.950, Student’s t-test). However, we noticed that both values were slightly higher in the samples collected during the early years. In more recent years, a similar COI has been discovered along the China-Myanmar border, as well as in previous years. Although the polyclonal proportion was higher in the early years compared to recent years (90.74% vs. 77.14%), the difference was not statistically significant (*P* = 0.055, Fisher’s exact test).

### Population diversity

The pairwise nucleotide diversity (*π*), number of haplotypes (*Nh*), number of different alleles (*Na*), number of effective alleles (*Ae*), and expected heterozygosity (*He*) for each population are shown in Table 1. Of these, the *π* value of WM was significantly higher than that of NEM2018 (*P* < 0.0001, Student’s t-test). For the sites along the eastern Myanmar border, *π* values of samples collected in 2011 varied slightly, ranging from 0.348 to 0.388, which were significantly higher than that in the NEM2018 parasites (*P* < 0.0001, Student’s t-test). At the China-Myanmar border, nucleotide diversity in parasite populations showed a noticeable reduction from 0.388 in 2011 (WC2011) to 0.224 in 2018 (NEM2018) (*P* < 0.0001, Student’s t-test). Remarkably, 100 (74.62%) unique haplotypes were detected in these 134 samples with monoclonal and biclonal infections (Table 1). Likewise, the WC, SM, and WT parasite populations harbored the greatest number of haplotypes in 2011. A pronounced decrease in the expected heterozygosity was observed at the China-Myanmar border from 0.363 in 2011 to 0.22 in 2018 (*P* < 0.0001, Student’s t test) (Table 1). Collectively, the *P*. *vivax* populations from the western GMS in 2011 were highly diverse without statistically significant differences among sites, while the diversity showed considerable reductions in recent years (2018).

**Table 1:** Genetic diversity of *P*. *vivax* populations in the GMS.

Population	N	*π* ± SD	*Nh*	*Na* ± SE	*Ae* ± SE	*He* ± SE
**WC2011**	23	0.388 ± 0.046	23	2.000 ± 0.000	1.624 ± 0.046	0.363 ± 0.020
**SM2011**	25	0.378 ± 0.044	24	1.949 ± 0.036	1.593 ± 0.041	0.354 ± 0.020
**WT2011**	23	0.348 ± 0.043	23	1.923 ± 0.057	1.577 ± 0.056	0.331 ± 0.026
**NEM2018**	49	0.224 ± 0.036	20	1.923 ± 0.043	1.347 ± 0.033	0.220 ± 0.026
**WM2018**	14	0.384 ± 0.050	10	1.897 ± 0.049	1.615 ± 0.050	0.352 ± 0.025
**Total**	134	0.363 ± 0.034	100	2.000 ± 0.000	1.616 ± 0.042	0.364 ± 0.018

N, number of samples; *π*, pairwise nucleotide diversity; *Nh*, number of haplotypes; *Na*, number of different alleles; *Ae*, number of effective alleles; *He*, expected heterozygosity; SD, standard deviation; SE, standard error. WC, western China; NEM, northeastern Myanmar; WM, western Myanmar; SM, southern Myanmar; WT, western Thailand.

We used the SMM and IAM models to estimate the effective population size (*Ne*) of *P*. *vivax* populations in the GMS. When the new *P*. *vivax* mutation rate was used, the *Ne* was largest in WC2011, followed by WM2018, and the smallest *Ne* was found in NEM2018 (Table 2), consistent with the results of nucleotide diversity.

**Table 2:** Effective population size (*Ne*) of the *P*. *vivax* populations was estimated using the SMM and IAM models.

Populations	SMM	95%CI	IAM	95%CI
**WC2011**	328648	172695–6656620	255771	134401–5180534
**SM2011**	313345	164654–6346662	245955	129242–4981706
**WT2011**	248317	130484–5029547	202594	106457–4103444
**NEM2018**	144447	75903–2925707	126594	66522–2564103
**WM2018**	310031	162912–6279531	243811	128116–4938272
**Total**	330389	173610–6691874	256879	134982–5202973

Mutation rate (*μ*) of *P*. *vivax* of 5.57 × 10^−7^ (95% confidence interval: 2.75 × 10^−8^, 1.06 × 10^−6^) was used. SMM, stepwise mutational model; IAM, infinite allele model. WC, western China; NEM, northeastern Myanmar; WM, western Myanmar; SM, southern Myanmar; WT, western Thailand.

### Genetic variation and differentiation

**Fig 4:**
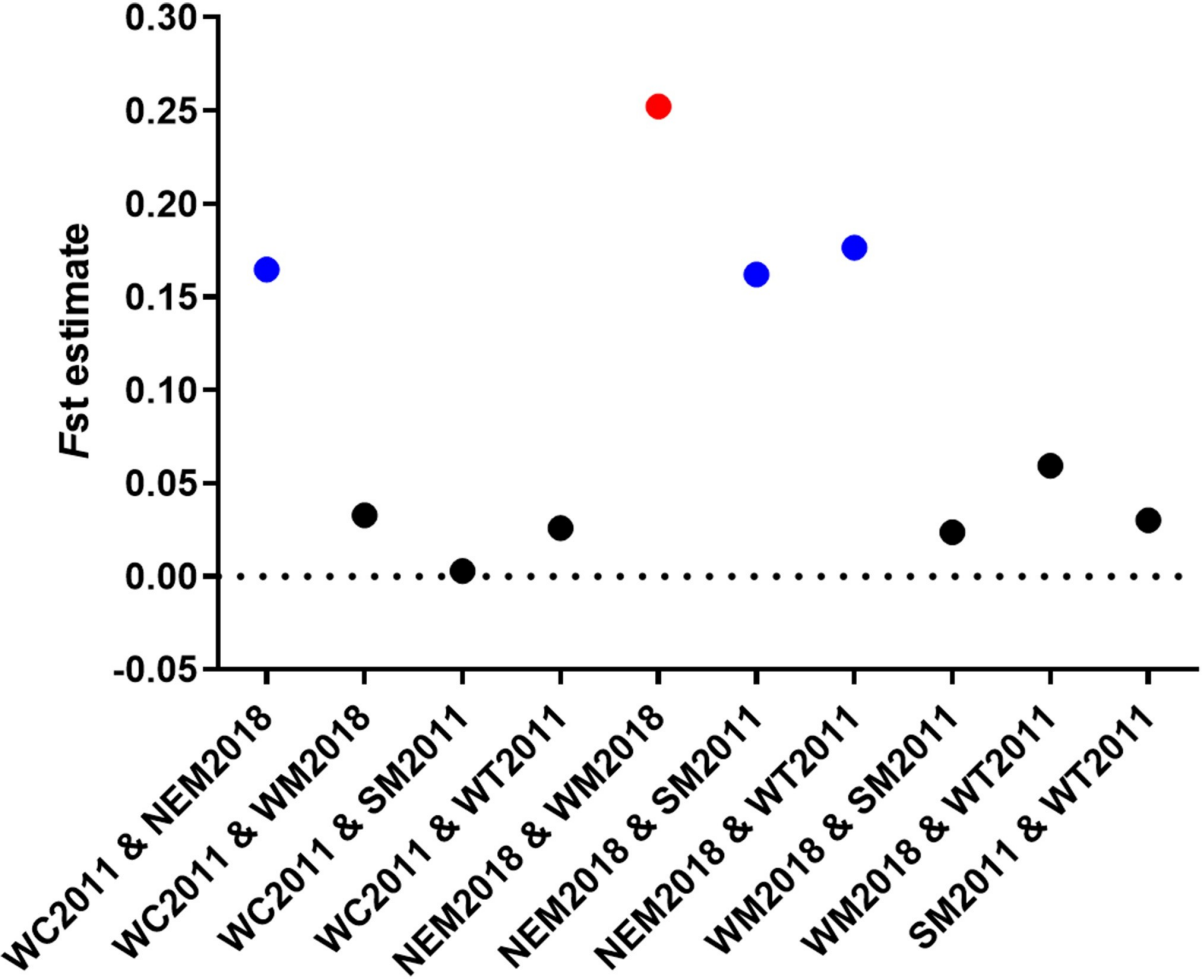
Pairwise comparison of *F*_ST_ among *P*. *vivax* populations in the GMS. Red, blue and black points represent high (*F*_ST_ ≥ 0.25), moderate (0.15 < *F*_ST_ < 0.25), and low genetic differentiation (*F*_ST_ ≤ 0.15), respectively.

AMOVA analysis revealed that the majority of the variations (87%) in *P*. *vivax* was found within populations in the GMS rather than between populations (S3 Fig). Mantel testing was conducted to determine the correlation between pairwise genetic and geographic distances of *P*. *vivax* populations, which showed a correlation coefficient *r*^*2*^ = 0.1693 (*P* = 0.140) (S4 Fig). To further clarify if genetic differentiation of the *P*. *vivax* populations existed in the GMS, Wright’s fixation index *F*_ST_ was estimated between each pair of the populations (Fig 4). Pairwise comparison determined the *F*_ST_ values ranging from 0.003 to 0.252. It is noteworthy that the NEM2018 parasite populations had moderate to high genetic differentiation from all other populations, with an *F*_ST_ value ranging from 0.162 to 0.252 (Fig 4). In particular, although the NEM2018 and WC2011 were essentially from the same area, the two longitudinal populations still had moderate genetic differentiation (*F*_ST_ = 0.165). In 2018, *P*. *vivax* from NEM and WM showed the highest genetic differentiation (*F*_ST_ = 0.252). In contrast, the other six pairwise comparisons did not show much genetic differentiation, with *F*_ST_ less than 0.15 (Fig 4). A global genetic differentiation analysis comparing *P*. *vivax* populations from the GMS, South America, Africa, and South Asia showed *F*_ST_ values ranging from 0.231 to 0.419 (S4 Table), which supports substantial division of the global *P*. *vivax* populations.

### Population structure

**Fig 5:**
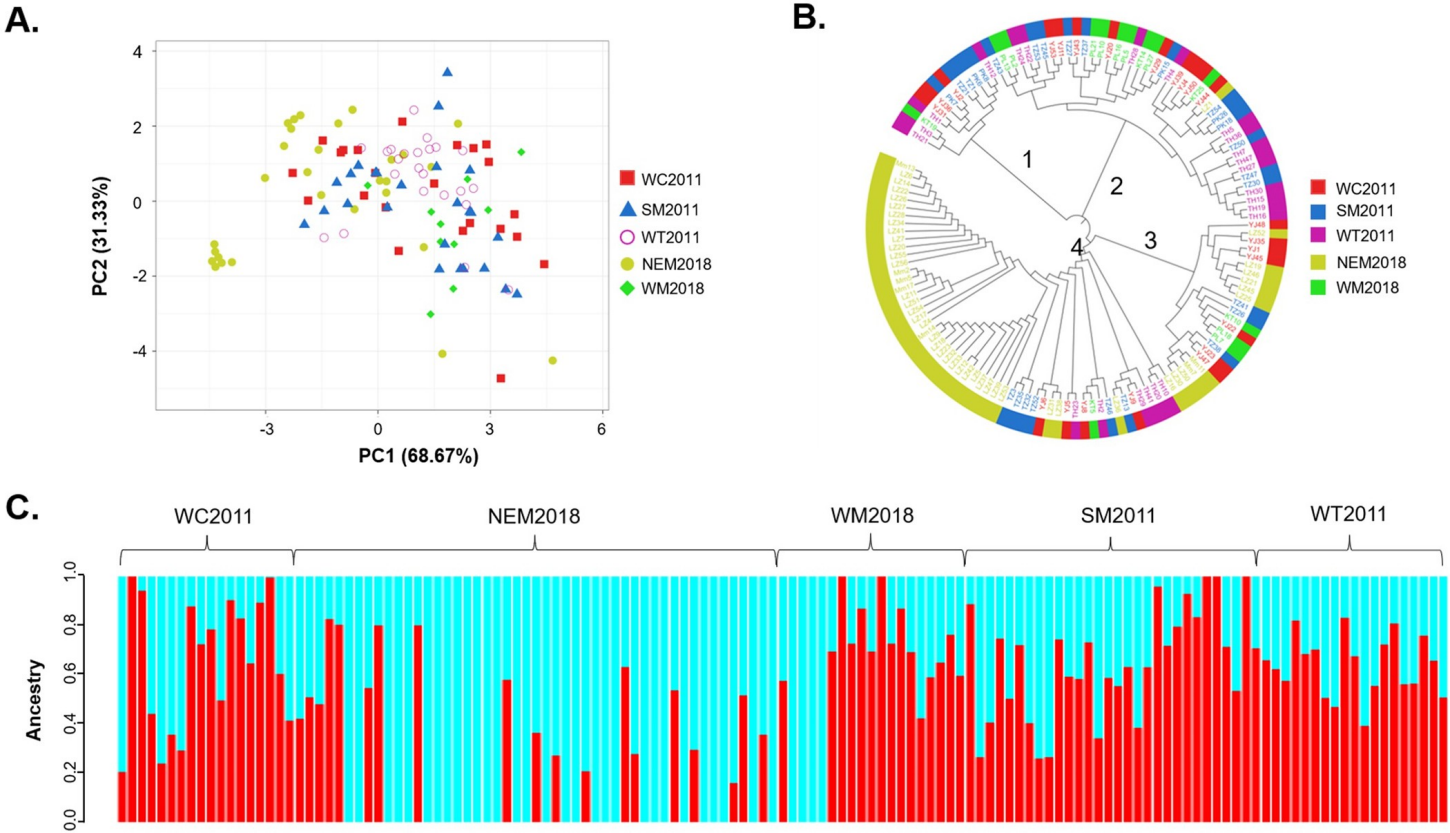
Clustering patterns of *P*. *vivax* isolates collected from the GMS. (A) Principal component analysis of the GMS samples. (B) Phylogenetic analysis of *P*. *vivax* isolates based on neighbor-joining method. (C) ADMIXTURE analysis for K = 2. Vertical bars indicate individual *P*. *vivax* haplotypes, and colors represent individual assignments to inferred clusters.

We further compared the *P*. *vivax* populations from different regions using PCA, phylogeny, and ADMIXTURE analysis. The PCA analysis revealed that the 39-SNP barcode sufficiently differentiated the global populations (S5A Fig), indicating that the removal of three SNPs (total MAF < 0.05) did not affect the overall effectiveness of the 42-SNP barcode. The main cluster comprised parasites from the GMS, which were separated from populations in Africa, South Asia, and South America (S5B Fig). We then used the 39-SNP barcode to perform PCA on *P*. *vivax* populations within the GMS. The results showed that the top two principal components accounted for 68.67% (PC1) and 31.33% (PC2) of the total variability. The NEM2018 population formed several clusters, and two clusters were well separated from other *P*. *vivax* populations from the GMS (Fig 5A). However, the rest of the populations, consisting of samples collected in 2011 and from western Myanmar in 2018, could not be confidently separated, suggesting close genetic relationships.

Phylogenetic analysis used to infer the genetic relationships among populations in the GMS identified four large clusters of parasites in the neighbor-joining tree (Fig 5B). Although each cluster contained parasites from all four sample collections, Cluster 4 included most of the NEM2018 samples. In particular, the NEM2018 samples in Cluster 4 formed a distinct clade, which contained three closely related main subclades. In a global analysis, the GMS population was also clearly distinguished from other populations from South Asia, Africa, and South America (S6 Fig).

The ADMIXTURE analysis of the western GMS samples revealed the presence of multiple subpopulations. Using the 39 SNPs with MAF above 0.05, we found that the lowest cross-validation error was at K = 2, which is considered the optimal number of populations (S7 Fig). At K = 2, all five parasite populations from GMS had admixed haplotypes and clustered into blue and red sub-populations (Fig 5C). The genetic structure patterns of four parasite populations, WC2011, SM2011, WT2011, and WM2018, were very similar, suggesting that many of these parasites had shared ancestry. In contrast, the NEM2018 population was genetically distinctive, with the blue sub-population dominated, which may reflect a process of population replacement.

## Discussion

The 42-SNP barcode with SNPs spanning all 14 chromosomes of the *P*. *vivax* genome was able to differentiate *P*. *vivax* populations among South Asia, Africa, and South America [27]. We utilized the Agena Bioscience MassARRAY System, a cost-effective and high-throughput platform that is compatible with the multiplex PCR design [57], to genotype 315 *P*. *vivax* parasite isolates collected from the western GMS using the 42 SNPs, with a 92% success rate. Failed samples were likely due to low DNA concentrations extracted from single blood spots on filter paper. This study extended the usability of the 42-SNP barcode to differentiate *P*. *vivax* populations from different continents, including Southeast Asia, despite removing three SNPs with MAF less than 0.05, which further validates the 42-SNP barcode as an effective way to track parasite origins to continents.

The COI provides valuable insights into disease transmission and epidemiology. Many methods estimate COI based on PCR amplification of one or more genetic loci with insertion/deletion polymorphisms, such as the *P*. *vivax* merozoite surface protein genes (*pvmsp3α* and *pvmsp3β*) and circumsporozoite protein [58–62]. In comparison, genotyping multiple loci is more accurate for detecting multiclonal infections. Our analysis revealed an average of 88.3% polyclonal *P*. *vivax* infections in the western GMS, comparable to the eastern GMS (e.g., 92% in Cambodia based on the analysis of a 100-SNP barcode) [45]. In this regard, the proportion of polyclonal infections in *P*. *vivax* samples in the GMS are much higher than in South Asia (41.2–79.0%), South America (52.8%), and Africa (39–60%) [27,32,63]. In contrast, analysis of similar *P*. *vivax* populations in the GMS by genotyping microsatellites only detected 14.6–30.7% as multiclonal infections [39, 40], further supporting the increasing power of the SNP barcode in detecting mixed infections. Within the western GMS, *P*. *vivax* samples from western Myanmar showed significantly higher COI than in northeastern Myanmar, reflecting higher *P*. *vivax* transmission intensity in western Myanmar, as we previously noted [64]. A notable observation from the analysis of longitudinal samples from northeast Myanmar is the significantly increased proportions of monoclonal infections from 2011 to 2018, which may reflect the more extensive control efforts delivered to this region. The northeast Myanmar site bordering China’s Yunnan Province is located in an extended buffer zone, where Yunnan’s malaria control program has implemented intensive malaria control efforts under its “3 + 1” strategy [65,66].

The *P*. *vivax* populations in the western GMS displayed moderate to high levels of genetic diversity, with the *π* values ranging from 0.224 to 0.388. Previous studies of GMS *P*. *vivax* populations using microsatellite markers identified *He* values of 0.66–0.86 [40,67,68], much higher than we found in this study (0.22–0.36). This difference may be related to the different genotyping methods used. Nevertheless, the effective population size *Ne* at the China-Myanmar border showed a ~ twofold decrease over seven years, consistent with the decreased genetic diversity of the parasite population. In comparison, the genetic diversity in the 2018 western Myanmar population was comparable to that in other regions of Myanmar in 2011, suggesting that control efforts did not significantly reduce parasite populations in the western border of Myanmar. Notably, one limitation of the study is that the China-Myanmar border samples were collected from two sites located on different sides of the border, although they are adjacent and connected by a major port facilitating migration.

F-statistics and population structure analysis suggested a relatively panmictic *P*. *vivax* population in the western GMS in 2011 before regional malaria elimination was on the agenda [33]. The higher within-population than between-population genetic variances also support this conclusion. However, parasites collected in 2018 from the China-Myanmar border formed distinct clades or clusters, suggesting increasing differentiation of *P*. *vivax* populations in the GMS in recent years. This is consistent with the population genomics analysis of *P*. *vivax* samples collected from northeast Myanmar, which were genetically distinct from other parts of the GMS [30]. Within the clusters, there were many closely related parasites suggesting results of clonal expansion. Such speculation is consistent with the *P*. *vivax* epidemiology in northeast Myanmar, where a vivax malaria outbreak occurred in 2016 [35]. The *P*. *vivax* population structure and dynamics in northeast Myanmar exhibited initial contraction of the parasite population and subsequent rapid clonal expansion, akin to the parasite population dynamics observed during the malaria pre-elimination phase in Malaysia [69].

Analyses of global *P*. *vivax* populations using microsatellites, antigens, and genome-wide SNPs have all identified strong geographic population structures between continents [12,18,19,70]. Similarly, the 42-SNP barcode captured differences between *P*. *vivax* populations from Africa, South America, South Asia, and Southeast Asia, but its ability to differentiate local populations within the GMS is limited. There are several possible explanations. Firstly, differences in selective pressure (human hosts, mosquito vectors, ecological environment, and malaria control measures) are strong at the global level but weak at the local level, resulting in less differentiated local parasite populations. The primary vectors *An*. *dirus* and *An*. *minimus* are widely distributed across the GMS [34]. As the GMS is pursuing regional malaria elimination, major malaria control measures, such as long-lasting insecticide-treated bed nets and indoor residual spraying, have been extensively deployed, and chloroquine/primaquine remains the frontline treatment for *P*. *vivax* malaria [34]. Thus, these factors may not have imposed strong region-specific selection on *P*. *vivax* population in the GMS. Secondly, parasite populations in the GMS were more or less panmictic due to the large effective population sizes, geographic connectivity, and considerable mixing resulting from extensive human migration [7,71]. Economic development in the GMS has facilitated large-scale human mobility, including cross-border labor migration, enabling transnatinal malaria introduction and parasite population mixing [72, 73]. Thirdly, the 42 SNP alleles in the barcode were not informative for the GMS parasite populations, as they were selected based on *F*_ST_ values among global populations. Accordingly, after analyzing the *P*. *vivax* genomes from different regions of the GMS, another panel of SNPs was selected as local markers to differentiate parasite populations within this region [30], which awaits future validation.

## Conclusion

Despite notable progress in malaria prevention and control, vivax malaria continues to present a significant challenge in the GMS region, particularly in Myanmar. To assess the effectiveness of ongoing malaria control efforts, it is crucial to have a genotyping tool that can offer insights into the genetic diversity, relatedness, and transmission patterns of vivax malaria populations. Analysis of *P*. *vivax* populations in the western GMS using a global 42-SNP barcode found more homogeneous parasite populations a decade ago but a less diverse and more differentiated parasite population from the China-Myanmar border in recent years. However, the 2018 western Myanmar parasite population did not show substantial changes in genetic diversity and population structure, stressing the need for strengthened malaria control efforts in this region. Notably, although the chronological changes observed in the different sites were consisitent with the changes in malaria transmission intensity, we cannot exclude potential influences of sample collection bias since all samples were convenience samples from acute vivax patients. The 42-SNP barcode has limitations in differentiating *P*. *vivax* populations across the GMS, requiring an extended barcode tailored to local parasite populations.

## Supporting information

S1 Fig Mass spectrometry peaks of quality control samples were detected by using Typer 4.0 software. (TIF)

S2 Fig The minor allele frequency of 42 SNPs per sub-region. (TIF)

S3 Fig Analysis of molecular variance of *P*. *vivax* isolates obtained from the GMS. (TIF)

S4 Fig Mantel test of *P*. *vivax* isolates obtained from the GMS. (TIF)

S5 Fig Principal coordinate analysis of the global sample sites. (A) The lessened barcode of 39 SNPs showed same ability to distinguish *P*. *vivax* populations as 42-SNP barcode. (B) Parasites from the GMS made up the main cluster, separated from Africa, south Asia and South America populations.
(TIF)

S6 Fig The phylogenetic analysis of the global sample sites. (TIF)

S7 Fig K-value (K = 2) had the lowest cross validation error (CV-error). (TIF)

S1 Table 42-SNP molecular barcode and primers. (DOCX)

S2 Table Minor allele frequency (MAF) of *P*. *vivax* populations. Red font indicates that the total MAF value is less than 0.05. WC, western China; NEM, northeastern Myanmar; WM, western Myanmar; SM, southern Myanmar; WT, western Thailand.
(DOCX)

S3 Table Clinical *P*. *vivax* samples were included in this study. (DOCX)

S4 Table Pairwise comparison of *F*_ST_ among global *P*. *vivax* populations. (DOCX)

S1 Dataset Barcodes for the 315 clinical *P*. *vivax* isolates in the GMS. The top panel shows the assays number its corresponding reference (REF.) or alternate allele (ALT.) and chromosome (Chrom.) position. The reference allele is shown in white, the alternate allele in gray, polygenomic genotypes with both alleles are labeled N and is shown in blue, missing SNPs are labeled X and is shown in red.
(XLSX)
