# Supplementary figures and images for: Plasmodium vivax populations in the western Greater Mekong Subregion evaluated using a genetic barcode
Source: PLoS Negl Trop Dis. 2024 Jul 3;18(7):e0012299. doi: 10.1371/journal.pntd.0012299 (PMC11251639; doi:10.1371/journal.pntd.0012299)

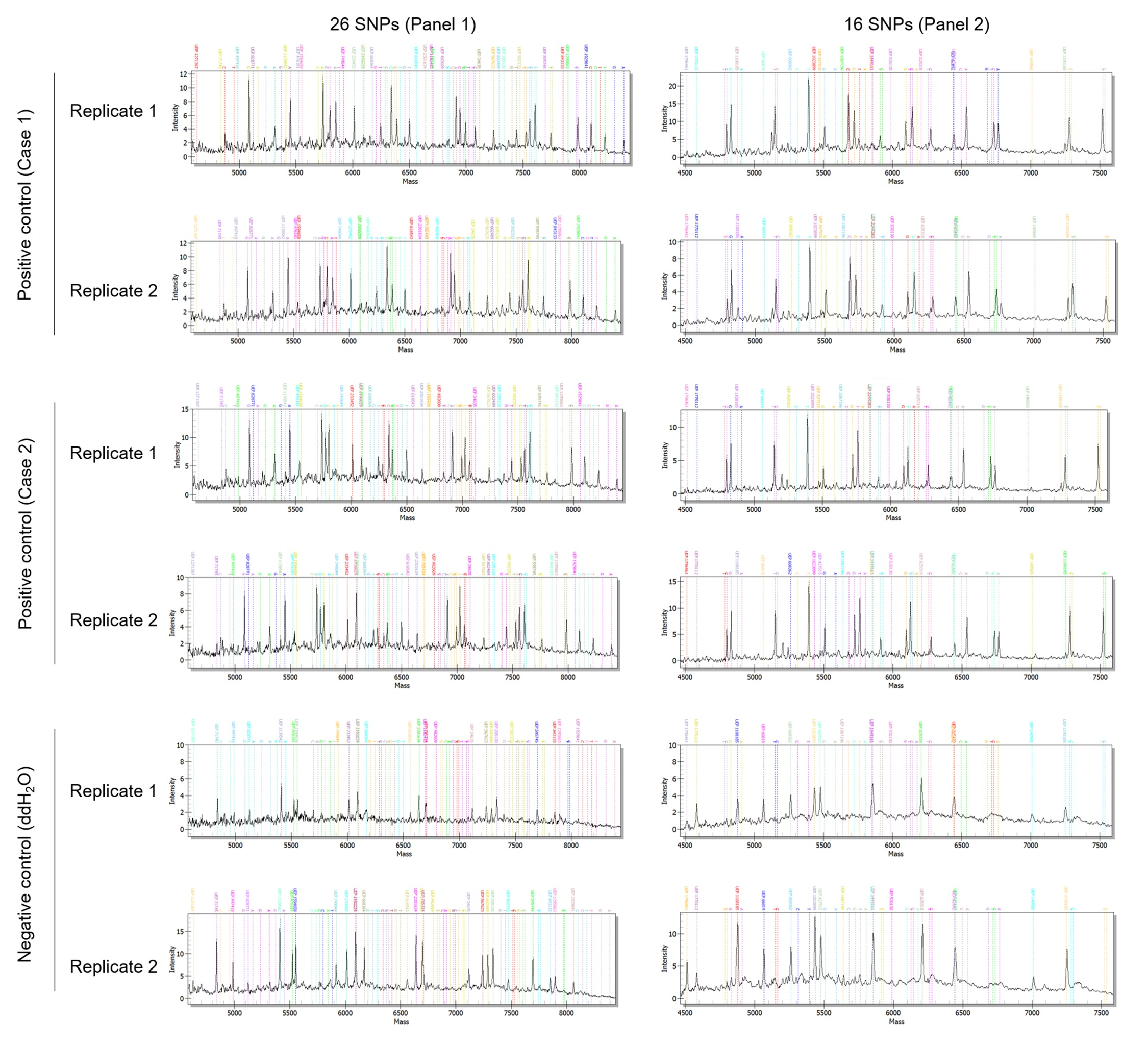

Supplement: S1 Fig — (TIF) [file pntd.0012299.s001.tif]

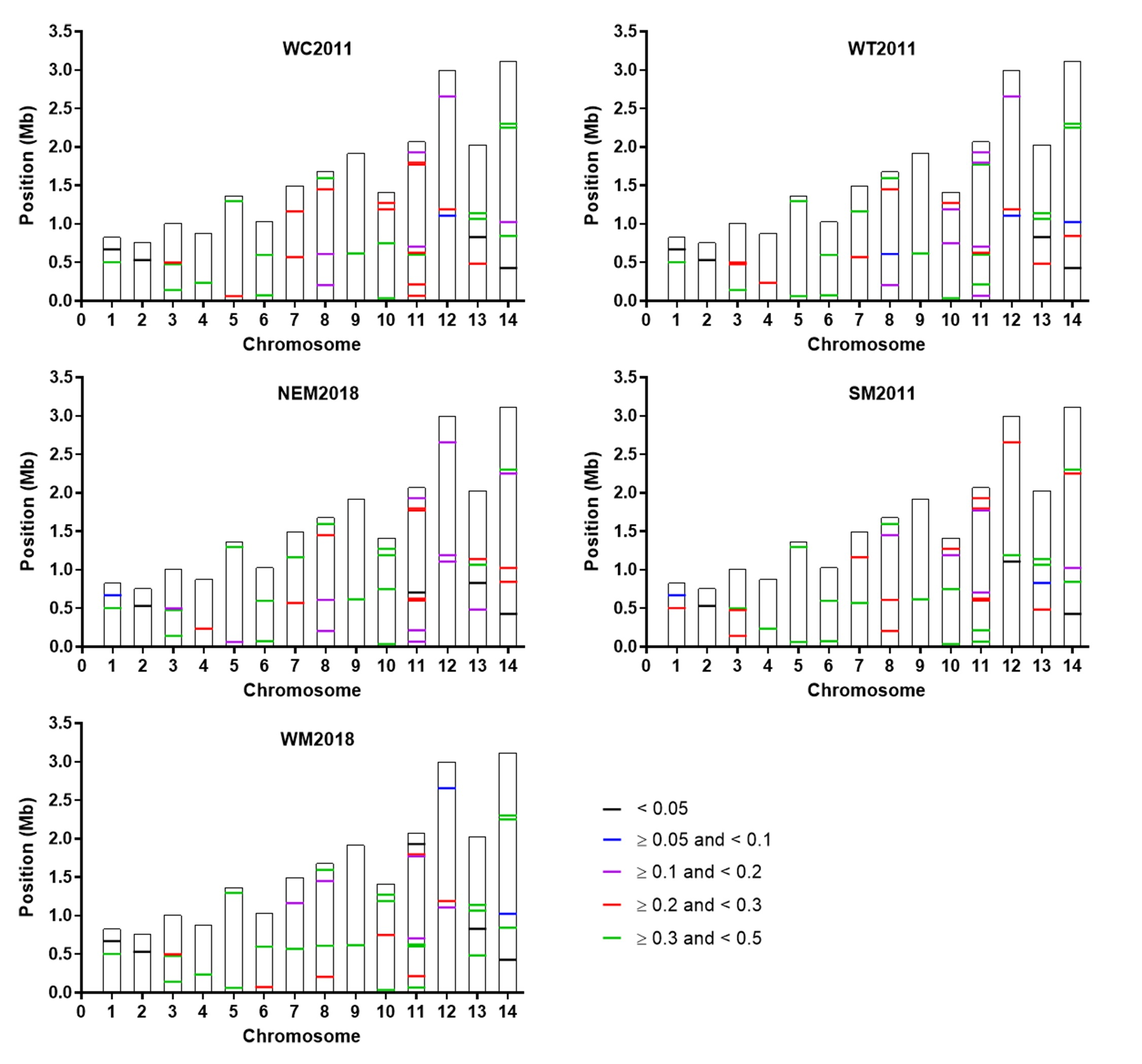

Supplement: S2 Fig — (TIF) [file pntd.0012299.s002.tif]

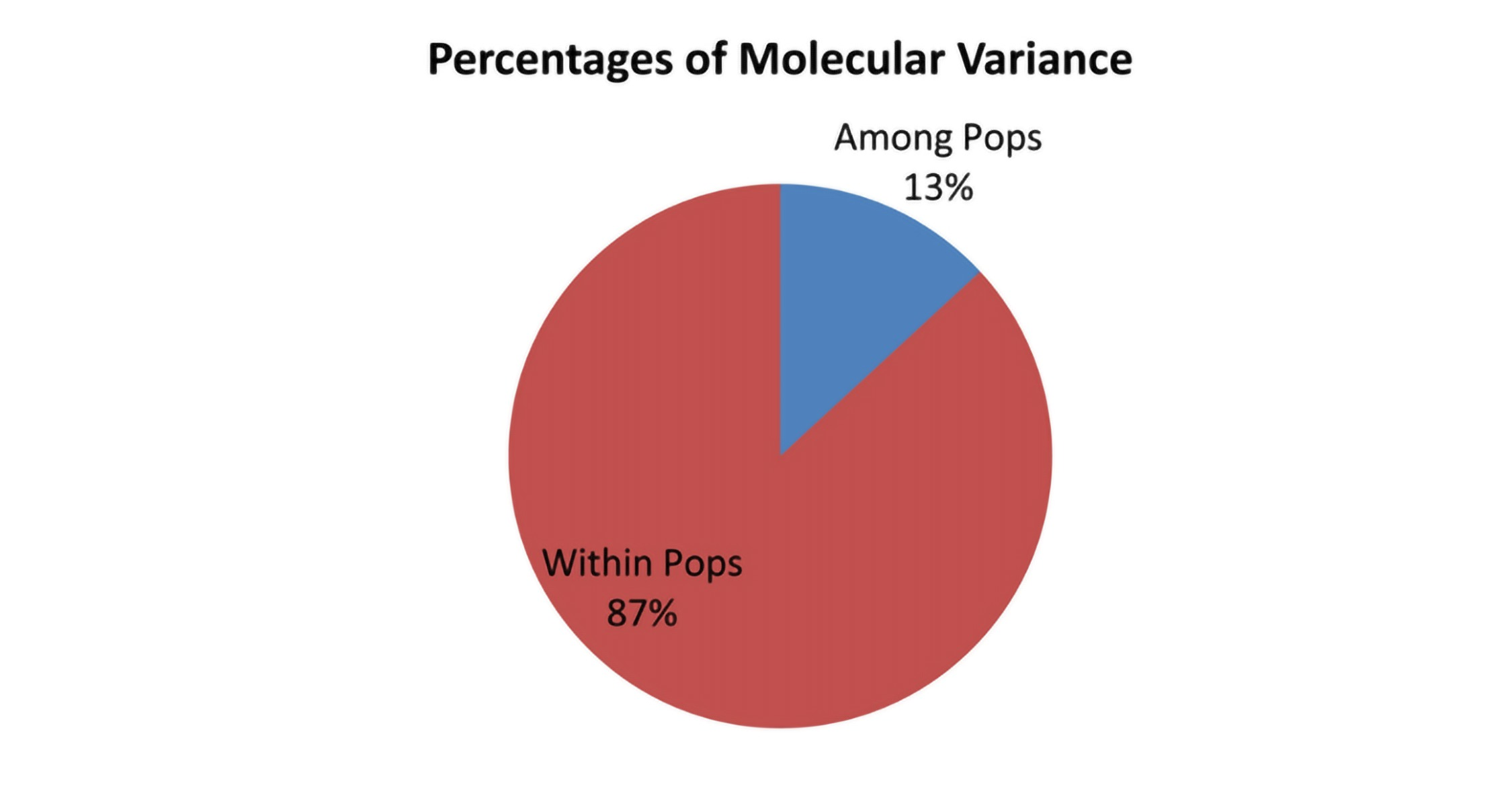

Supplement: S3 Fig — (TIF) [file pntd.0012299.s003.tif]

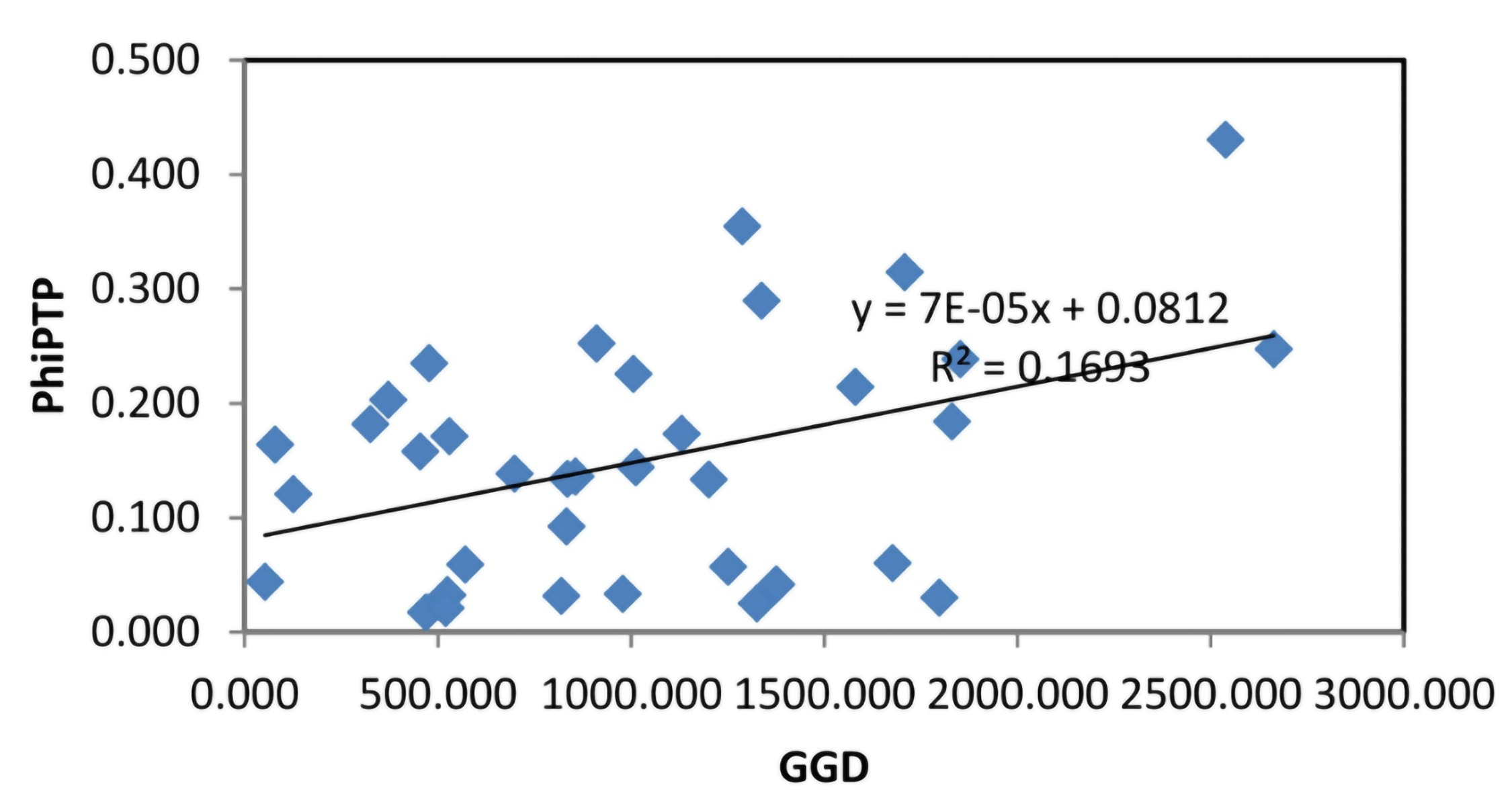

Supplement: S4 Fig — (TIF) [file pntd.0012299.s004.tif]

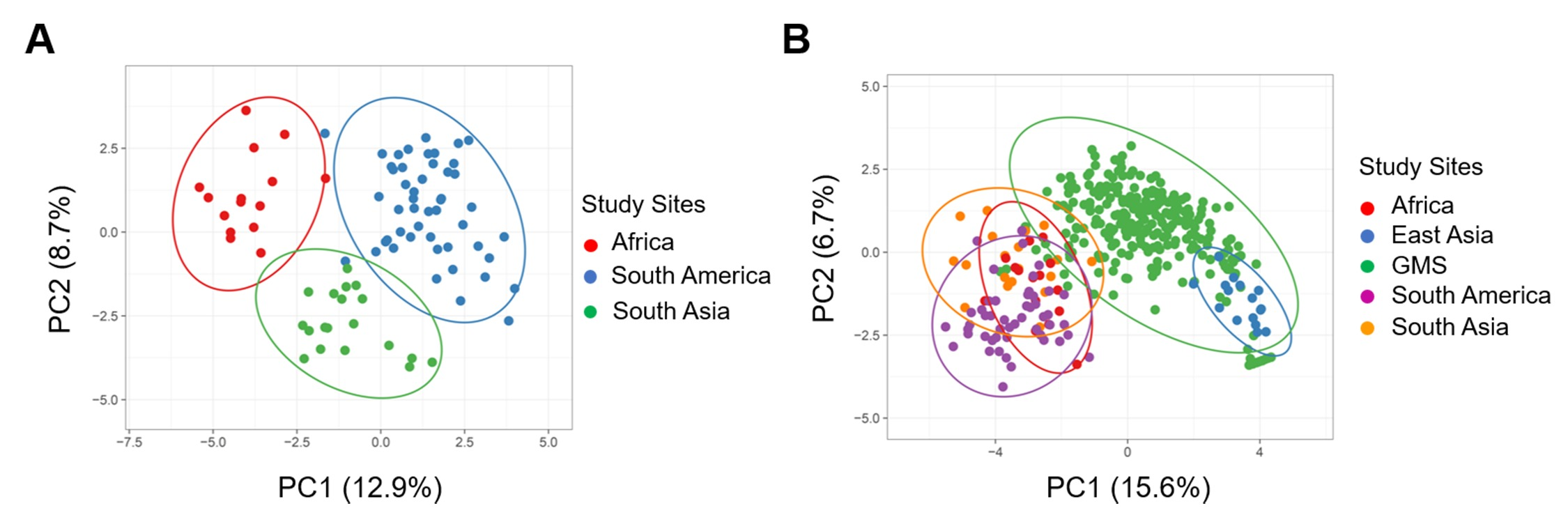

Supplement: S5 Fig — (A) The lessened barcode of 39 SNPs showed same ability to distinguish P. vivax populations as 42-SNP barcode. (B) Parasites from the GMS made up the main cluster, separated from Africa, south Asia and South America populations. (TIF) [file pntd.0012299.s005.tif]

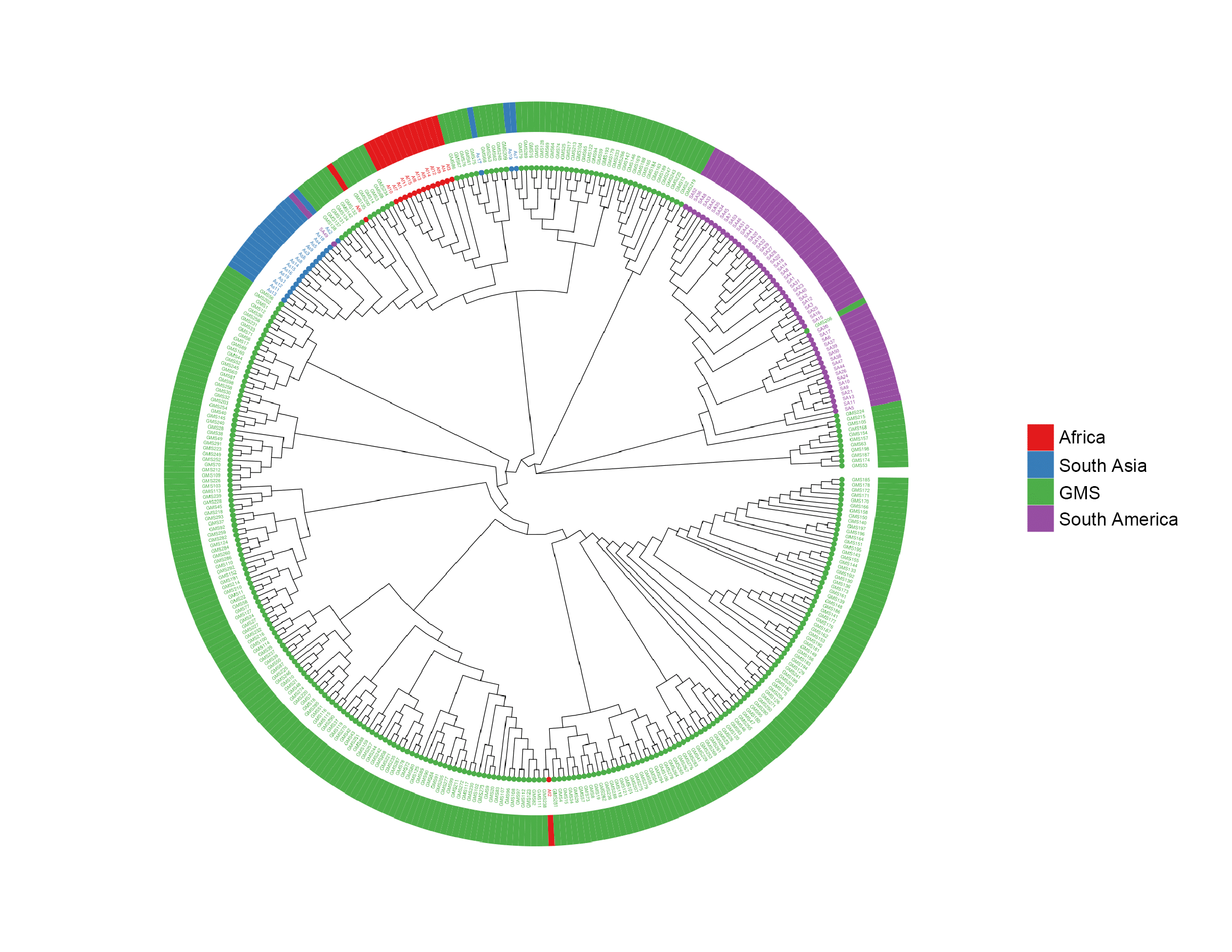

Supplement: S6 Fig — (TIF) [file pntd.0012299.s006.tif]

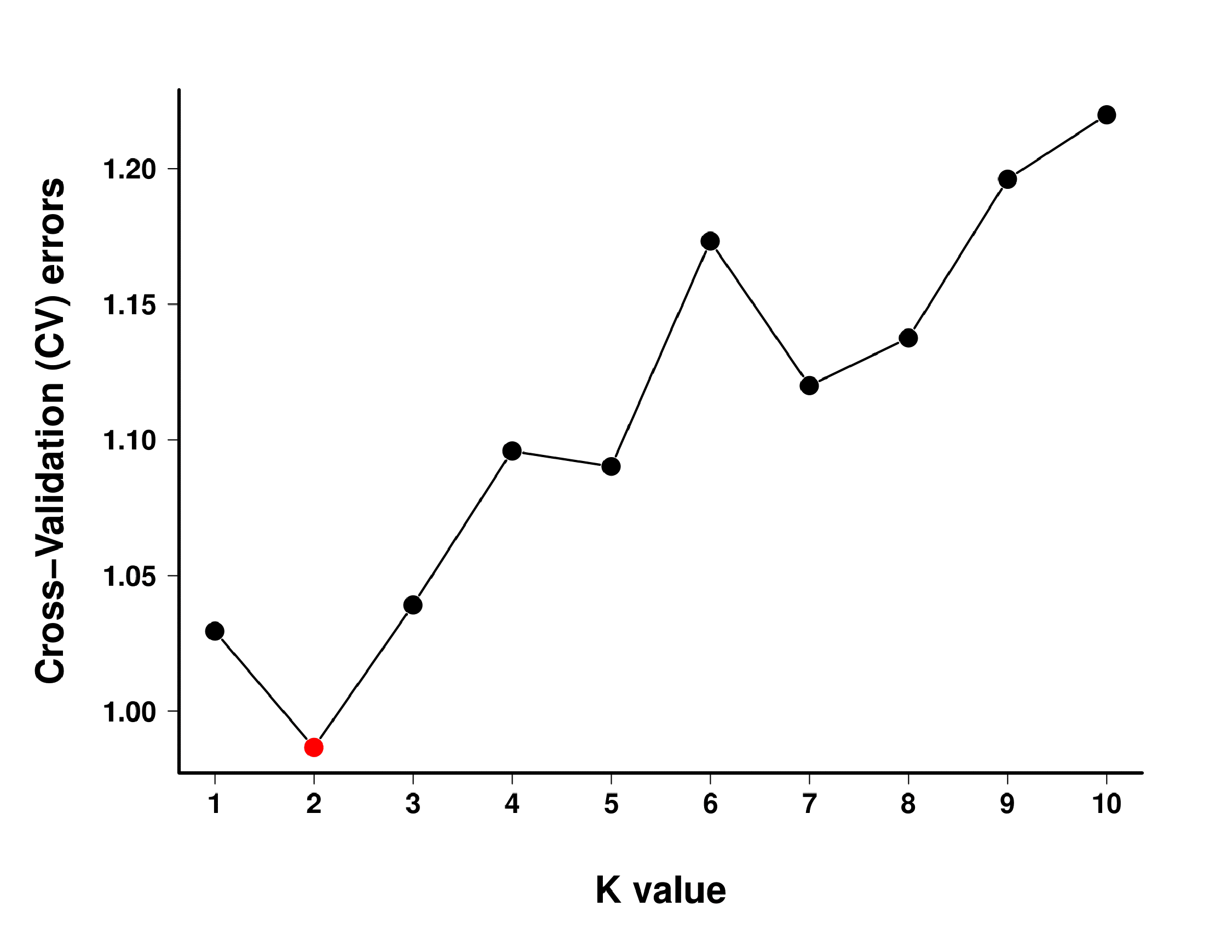

Supplement: S7 Fig — (TIF) [file pntd.0012299.s007.tif]
