# Supplementary material for: Plasmodium vivax populations in the western Greater Mekong Subregion evaluated using a genetic barcode
Source: PLoS Negl Trop Dis. 2024 Jul 3;18(7):e0012299. doi: 10.1371/journal.pntd.0012299 (PMC11251639; doi:10.1371/journal.pntd.0012299)
Supplement: S1 Table — (DOCX) [file pntd.0012299.s008.docx]

**S1 Table. 42-SNP molecular barcode and primers.**

| **No.** | **Group-SNPs** | **Position** | **1st-PCRP** | **2nd-PCRP** | **UEP_SEQ** |
| --- | --- | --- | --- | --- | --- |
| **1** | P1-1 | 502333 | ACGTTGGATGAAGTGAGTCGCATCTAGCAG | ACGTTGGATGCCTACAGAACCTACTACGTG | gtaaGTGAGGGACGACTTACAAGA |
| **2** | P1-2 | 668364 | ACGTTGGATGTAGCTTGCTCCTGTTCTTGC | ACGTTGGATGTCCAACCGGGTGGGTAACAT | aGGGTGGGTAACATCGTCTC |
| **3** | P1-5 | 476181 | ACGTTGGATGGGACCTGATTCGGAAAATGC | ACGTTGGATGAGCCTTATCCACTCATGCTC | AGCGTCTCTAGCGGAAAT |
| **4** | P1-6 | 497418 | ACGTTGGATGAAACCGTAGGGTTGCAAAGC | ACGTTGGATGGCTGTTATGCGGTAGTTAGG | GGTAGTTAGGAGGCAC |
| **5** | P1-7 | 233452 | ACGTTGGATGAGGTGAAGTCAGTTCGAAGG | ACGTTGGATGAGAGGATAACATGGGCAGAG | ccccCGTTAGGAGTCCTCCA |
| **6** | P1-9 | 1295863 | ACGTTGGATGGAATGCGCCAGTGTTATAGG | ACGTTGGATGGGAGGGTTCCATTTACTGTG | tcctgTTAGATCCCCTCTAAGGACTG |
| **7** | P1-10 | 71745 | ACGTTGGATGGCGAGAAAAATTCGCACCAC | ACGTTGGATGCGCAAGTTTGTTTAGCCTCC | TTTAGCCTCCTCGGTG |
| **8** | P1-11 | 595745 | ACGTTGGATGCAAAGAACACGTCGATCAGG | ACGTTGGATGTAGTACCTCATGCGGTCATC | tcagGCGGTCATCAGATGTGTCAAT |
| **9** | P1-12 | 567623 | ACGTTGGATGGAAAAGGACCTCCTGAATGC | ACGTTGGATGACATTTGAGAAGCCTTCCCC | cccatCCTTCCCCAAATGAAGAGT |
| **10** | P1-13 | 1162433 | ACGTTGGATGTTACAGTGATCTCGGCTTCC | ACGTTGGATGAGGTGAACACCTACACAGAC | cCACAGACAACCTAAAAAAAGT |
| **11** | P1-14 | 205120 | ACGTTGGATGATATCGCGTATGCATGTGTG | ACGTTGGATGGAGCCAAACGTTGGCACAAA | acCGTTGGCACAAAAAATGTTTTC |
| **12** | P1-17 | 1594658 | ACGTTGGATGCGAACTGGTGAATCCTTCTG | ACGTTGGATGAGTTGACCACACTGACCAAG | CAAATGCTGGGAGTTTTA |
| **13** | P1-18 | 616543 | ACGTTGGATGAAATCGCGAGCCCTTTTTTC | ACGTTGGATGTTCCATCTGCCGAAATGACG | gggaCGAAATGACGAACAAGG |
| **14** | P1-19 | 34635 | ACGTTGGATGCCATAACATGTGGACATCCC | ACGTTGGATGACCTTCTGGATTGTCCTTTC | TGTAAATATAGCGTTTTGAAAGT |
| **15** | P1-20 | 749044 | ACGTTGGATGAACACCCGTGGTGGGCACAT | ACGTTGGATGGGACTGCAACTTTTGAAGGG | gggaCGGAGCGGATTCGCA |
| **16** | P1-22 | 1271367 | ACGTTGGATGAAAGTTTGCAGCCCCGTACC | ACGTTGGATGGCTGTTGAGAAGAAAGCCAC | GTGAAAGGTACGCCA |
| **17** | P1-25 | 602489 | ACGTTGGATGCCAAGGGCGCCATCATTTTG | ACGTTGGATGGAAGCCGCAAGACAGCATTC | ACAGCATTCCTTTAACAACAAAGT |
| **18** | P1-27 | 705724 | ACGTTGGATGGCACATGTGTAACAGTTGCG | ACGTTGGATGTCGGAAGGATGCCTAAATGG | tatcGCGCTCTCTGTGTATATG |
| **19** | P1-30 | 1929841 | ACGTTGGATGCCCCATTGTAAATCCCAACC | ACGTTGGATGTTTTTCCCCTGCACTAGCTG | ctgagGAATGCGTTTGATTGTTCTGG |
| **20** | P1-32 | 1190029 | ACGTTGGATGCAGAGTCGTCGCAACGATAA | ACGTTGGATGTCCTGGCAAAAGTTCTCCTG | aaccGCAGGCTGTTGCTCAT |
| **21** | P1-33 | 2656099 | ACGTTGGATGCAATCTAAGGGTGAGTCCTG | ACGTTGGATGGACCAAATTGCATGCCTTCG | atcggTGGGTCCCCCTTTAG |
| **22** | P1-34 | 482609 | ACGTTGGATGACTGCGTGCTCCAATTTGAC | ACGTTGGATGCAATCGCAGTTTGCCAAACG | acagGGGCACACGCGGCATAGT |
| **23** | P1-35 | 828771 | ACGTTGGATGGTTAGGGAGATAGTCCTACG | ACGTTGGATGAGGAGTTAGCCATGTGACTG | cATACACAACTGCTGCA |
| **24** | P1-37 | 1139542 | ACGTTGGATGGCTGGACAAGATGTTTTCCC | ACGTTGGATGGCTTCGATGGCGCTTCTATG | CTATGCCGCTTCGATACC |
| **25** | P1-39 | 843133 | ACGTTGGATGTGCTGTGCAAAGGTAGGTAG | ACGTTGGATGGGAGCTAATTTACCTTCCCG | tccTTTACCTTCCCGTTAATGTGTCG |
| **26** | P1-42 | 2301634 | ACGTTGGATGCCCGATTAGCAGCAAAAAAG | ACGTTGGATGCATGGATGTAACTTGACGGC | cccgaTGCCCATTTCGCCAAAG |
| **27** | P2-3 | 530130 | ACGTTGGATGGTCCAAATTGGCTGATTCGC | ACGTTGGATGTTATTGGAGTAGTCCTCCCG | acctcCTCCCTGTTCGAGCA |
| **28** | P2-4 | 140565 | ACGTTGGATGGCGCTAAAGTTTTTGGCCTC | ACGTTGGATGCTGATCAGTGGACGGGTAAA | GCAAAATAATCATCACAGTAATC |
| **29** | P2-8 | 62892 | ACGTTGGATGGGAACTATCCATTAAGTCTTC | ACGTTGGATGGACCAACAGATCACTGTATG | CAGATCACTGTATGAAGATAC |
| **30** | P2-15 | 608362 | ACGTTGGATGTATTTCCAGTTCCAGGGCAC | ACGTTGGATGTCATTGTACCCCCCATTTGC | tAGGAACAAGACGACGA |
| **31** | P2-16 | 1449111 | ACGTTGGATGCGTGCGTATGTTCATGCGTA | ACGTTGGATGAGGGCGTTCTTCAAAACCTG | ggCAAAACCTGCCGAAAGG |
| **32** | P2-21 | 1190185 | ACGTTGGATGCTACTTCAGGATTAATAGCG | ACGTTGGATGGGACTTTTGTAGGTCCACAC | CTTTTCGATATCATTTTCAATTTT |
| **33** | P2-23 | 66819 | ACGTTGGATGATTTCCGCGGATGTGTTTCG | ACGTTGGATGAAAAGCTTCCTCGGATGCCC | CCTCCATTTCCGCGCAA |
| **34** | P2-24 | 215123 | ACGTTGGATGCGCCATGAGGAGAGATAAAC | ACGTTGGATGGCGCTTCCATCATTCCTCTG | gataATTCCTCTGGCAGAGGC |
| **35** | P2-26 | 625318 | ACGTTGGATGCGTTTCATTTTGCCCTTCCG | ACGTTGGATGTCAGACTAGTAGAGCAGACG | taaGCGAGGTAGAAAGAACC |
| **36** | P2-28 | 1770112 | ACGTTGGATGGGTGGAGTAAGCGGAAAAAC | ACGTTGGATGTGAGCGCTGAACTGGTCTAC | TGGTCTACACTGGCG |
| **37** | P2-29 | 1796461 | ACGTTGGATGCGCTCAGTCGTTTTTTGCAG | ACGTTGGATGAGTAGCCTCACTAATGGGTC | TCCCTTGCACAGCAG |
| **38** | P2-31 | 1108185 | ACGTTGGATGTCTGACGAGCAGCCACAAC | ACGTTGGATGAAACGCCCCCTTTGAGTATC | TTTGGGTGTGCCACTC |
| **39** | P2-36 | 1067343 | ACGTTGGATGATTCTGTCCACCGAGCTCAC | ACGTTGGATGTCTACGTCGGCATGGAAAAG | aAGCCAAAGGGGAAGAAG |
| **40** | P2-38 | 427515 | ACGTTGGATGCCTCCTTCTTCTGATTCGTG | ACGTTGGATGATGACCCGTACAGTAACGTC | GACCACCACGGTTAATAA |
| **41** | P2-40 | 1023899 | ACGTTGGATGATTTCTGCCCCTTCATGTGG | ACGTTGGATGTGGAAAAAGCGTCTTATCAG | cCGTCTTATCAGCCATGA |
| **42** | P2-41 | 2249283 | ACGTTGGATGACCTTCGCCTACAAGAACTC | ACGTTGGATGAGAACTCCACCTGCCTTATG | GCCTTATGTATTTCAGGGG |
