# Supplementary material for: Plasmodium vivax populations in the western Greater Mekong Subregion evaluated using a genetic barcode
Source: PLoS Negl Trop Dis. 2024 Jul 3;18(7):e0012299. doi: 10.1371/journal.pntd.0012299 (PMC11251639; doi:10.1371/journal.pntd.0012299)
Supplement: S2 Table — Red font indicates that the total MAF value is less than 0.05. WC, western China; NEM, northeastern Myanmar; WM, western Myanmar; SM, southern Myanmar; WT, western Thailand. (DOCX) [file pntd.0012299.s009.docx]

**S2 Table.** **Minor allele frequency (MAF) of *P. vivax* populations.**

| **Assay** | **SNPs** | **Chrom.** | **MAF (Total)** | **MAF (WC)** | **MAF (NEM)** | **MAF (WM)** | **MAF (SM)** | **MAF (WT)** |
| --- | --- | --- | --- | --- | --- | --- | --- | --- |
| **1** | 502333 | 1 | 0.4209 | 0.37955 | 0.4621 | 0.3305 | 0.2812 | 0.4444 |
| **2** | 668364 | 1 | 0.0556 | 0.0462 | 0.0769 | 0.0164 | 0.06875 | 0.02273 |
| **3** | 530130 | 2 | 0 | 0 | 0 | 0 | 0 | 0 |
| **4** | 140565 | 3 | 0.4868 | 0.34883 | 0.3175 | 0.3917 | 0.277 | 0.4091 |
| **5** | 476181 | 3 | 0.3323 | 0.31265 | 0.4038 | 0.425 | 0.2125 | 0.2093 |
| **6** | 497418 | 3 | 0.2379 | 0.2427 | 0.1985 | 0.2373 | 0.3141 | 0.2209 |
| **7** | 233452 | 4 | 0.4712 | 0.36415 | 0.2962 | 0.4262 | 0.4733 | 0.2609 |
| **8** | 62892 | 5 | 0.2631 | 0.2849 | 0.1786 | 0.3051 | 0.3377 | 0.3182 |
| **9** | 1295863 | 5 | 0.4190 | 0.43055 | 0.4070 | 0.4516 | 0.375 | 0.4886 |
| **10** | 71745 | 6 | 0.4409 | 0.3507 | 0.4077 | 0.2627 | 0.3688 | 0.3636 |
| **11** | 595747 | 6 | 0.4210 | 0.4303 | 0.3798 | 0.4833 | 0.4295 | 0.4286 |
| **12** | 567623 | 7 | 0.2751 | 0.2872 | 0.2244 | 0.3390 | 0.3063 | 0.2791 |
| **13** | 1162433 | 7 | 0.3221 | 0.28768 | 0.4727 | 0.178 | 0.2 | 0.3 |
| **14** | 205120 | 8 | 0.2013 | 0.199 | 0.1962 | 0.2458 | 0.2063 | 0.1477 |
| **15** | 608362 | 8 | 0.1677 | 0.1778 | 0.1047 | 0.3051 | 0.2125 | 0.08889 |
| **16** | 1449111 | 8 | 0.2222 | 0.20908 | 0.2946 | 0.1417 | 0.15 | 0.25 |
| **17** | 1594658 | 8 | 0.4010 | 0.40903 | 0.3962 | 0.418 | 0.3333 | 0.4886 |
| **18** | 616543 | 9 | 0.3641 | 0.3644 | 0.3411 | 0.3818 | 0.4125 | 0.3222 |
| **19** | 34635 | 10 | 0.4601 | 0.44315 | 0.4881 | 0.4831 | 0.3014 | 0.5 |
| **20** | 749044 | 10 | 0.4335 | 0.3052 | 0.3855 | 0.2833 | 0.4187 | 0.1333 |
| **21** | 1190185 | 10 | 0.2732 | 0.26158 | 0.3095 | 0.3814 | 0.1849 | 0.1705 |
| **22** | 1271367 | 10 | 0.3035 | 0.2883 | 0.3598 | 0.3115 | 0.2375 | 0.2444 |
| **23** | 66819 | 11 | 0.2511 | 0.2511 | 0.1992 | 0.3525 | 0.325 | 0.1277 |
| **24** | 215123 | 11 | 0.2611 | 0.28378 | 0.1822 | 0.2661 | 0.3312 | 0.3556 |
| **25** | 602489 | 11 | 0.2772 | 0.30303 | 0.2308 | 0.3305 | 0.2438 | 0.407 |
| **26** | 625318 | 11 | 0.2780 | 0.28393 | 0.2519 | 0.3226 | 0.2885 | 0.2727 |
| **27** | 705724 | 11 | 0.0931 | 0.10844 | 0.03876 | 0.1271 | 0.14 | 0.1279 |
| **28** | 1770112 | 11 | 0.2138 | 0.22183 | 0.2248 | 0.1667 | 0.1625 | 0.3333 |
| **29** | 1796461 | 11 | 0.2350 | 0.22848 | 0.2227 | 0.2823 | 0.2812 | 0.1277 |
| **30** | 1929841 | 11 | 0.1298 | 0.1225 | 0.1163 | 0.0339 | 0.2375 | 0.1023 |
| **31** | 1108185 | 12 | 0.0965 | 0.094 | 0.1211 | 0.1441 | 0.03125 | 0.07955 |
| **32** | 1190029 | 12 | 0.2508 | 0.2607 | 0.1938 | 0.2881 | 0.3269 | 0.234 |
| **33** | 2656099 | 12 | 0.1603 | 0.15424 | 0.1628 | 0.07627 | 0.2188 | 0.1591 |
| **34** | 482609 | 13 | 0.2516 | 0.26825 | 0.1856 | 0.3571 | 0.275 | 0.2553 |
| **35** | 828771 | 13 | 0.0373 | 0.04196 | 0.01515 | 0.04762 | 0.0625 | 0.04255 |
| **36** | 1067196 | 13 | 0.4524 | 0.44998 | 0.4031 | 0.4831 | 0.4563 | 0.4574 |
| **37** | 1139542 | 13 | 0.4071 | 0.39868 | 0.2617 | 0.4098 | 0.4813 | 0.4419 |
| **38** | 427515 | 14 | 0.0176 | 0.01418 | 0.02326 | 0.008475 | 0.025 | 0 |
| **39** | 843133 | 14 | 0.3182 | 0.31778 | 0.2713 | 0.3017 | 0.4423 | 0.2558 |
| **40** | 1023899 | 14 | 0.1748 | 0.14028 | 0.2773 | 0.08475 | 0.1447 | 0.05435 |
| **41** | 2249283 | 14 | 0.3071 | 0.35175 | 0.185 | 0.4754 | 0.2792 | 0.4674 |
| **42** | 2301634 | 14 | 0.3984 | 0.41293 | 0.3015 | 0.4754 | 0.443 | 0.4318 |

Red font indicates that the total MAF value is less than 0.05. WC, western China; NEM, northeastern Myanmar; WM, western Myanmar; SM, southern Myanmar; WT, western Thailand.
