# Supplementary material for: Plasmodium vivax populations in the western Greater Mekong Subregion evaluated using a genetic barcode
Source: PLoS Negl Trop Dis. 2024 Jul 3;18(7):e0012299. doi: 10.1371/journal.pntd.0012299 (PMC11251639; doi:10.1371/journal.pntd.0012299)
Supplement: S3 Table — (DOCX) [file pntd.0012299.s010.docx]

**S3 Table. Clinical *P. vivax* samples were included in this study.**

| **Population** | **Initial samples** | **After filtering ^a^** | **Polyclonal infections ^b^** | **Monoclonal infections ^c^** | **Biclonal infections ^d^** |
| --- | --- | --- | --- | --- | --- |
| **WC2011** | 58 | 54 | 49 | 5 | 18 |
| **SM2011** | 80 | 73 | 70 | 3 | 22 |
| **WT2011** | 47 | 42 | 34 | 8 | 15 |
| **NEM2018** | 75 | 70 | 54 | 16 | 33 |
| **WM2018** | 55 | 52 | 50 | 2 | 12 |
| **Total** | 315 | 291 | 257 | 34 | 100 |

^a^ For quality filtration, individuals with more than 10% missing SNP calls were filtered out.

^b^ Samples carried one or more polymorphic sites.

^c^ Samples carried a single allele at all positions.

^d^ Samples displayed a single polymorphic site.
