# Supplementary material for: Plasmodium vivax populations in the western Greater Mekong Subregion evaluated using a genetic barcode
Source: PLoS Negl Trop Dis. 2024 Jul 3;18(7):e0012299. doi: 10.1371/journal.pntd.0012299 (PMC11251639; doi:10.1371/journal.pntd.0012299)
Supplement: S4 Table — (DOCX) [file pntd.0012299.s011.docx]

S4 Table. Pairwise comparison of *F*_ST_ among global *P. vivax* populations.

| Po**pulations** | **GMS** | **South America** | **Africa** |
| --- | --- | --- | --- |
| **GMS** |  |  |  |
| **South America** | 0.345 |  |  |
| **Africa** | 0.419 | 0.409 |  |
| **South Asia (Sri Lanka)** | 0.372 | 0.231 | 0.331 |
